# Supplementary material for: The role of migration barriers for dispersion of Proliferative Kidney Disease—Balance between disease emergence and habitat connectivity
Source: PLoS One. 2021 Mar 17;16(3):e0247482. doi: 10.1371/journal.pone.0247482 (PMC7968629; doi:10.1371/journal.pone.0247482)
Supplement: Attachment — Submitted filename: PONE-D-20-18486_comments.docx [file pone.0247482.s001.docx]

General Comments:

I have several major conceptual criticisms of this work, which I will expand upon below. I understand that word limits often restrict methods details, but the methods, as they are described in the submission, are not detailed enough to be repeated. In addition, the manuscript does not seem like it is ready for submission, as there are several formatting anomalies.

The methods of the study do not address the objective as it is outlined in the introduction: “The aim of the study was to investigate the role of a migration barrier in limitation of pathogen transport by migrating fish…”. These data are not generalizable to other diseases and the study design is incapable of addressing this objective. As is pointed out in the manuscript, PKD generally manifests in naïve fish, which are generally YOY. As is also pointed out in the manuscript, YOY brown trout generally do not migrate into tributary streams from larger bodies of water. So the study’s conclusion that “the benefit of restoration of the river corridor seems to exceed the risk of transmission of infectious agents” is based on an extremely biased data set because the fish that are migrating are the same ones that are least likely to succumb to, be susceptible to, or to release parasite spores due to PKD.

Another major flaw is that the study completely ignores the primary bryozoan hosts of *T. bryosalmonae*. There was no mention of any effort to confirm the presence of bryozoans in the Ehrenbach, which would be necessary for the complete life cycle of *T. bryosalmonae*, because spores released from infected fish cannot, as far as we know, ever infect other fish. If bryozoans are not present in the Ehrenbach, might the results have been dramatically different if bryozoans were there? On a similar note, the absence of parasite in fish is not necessarily an accurate indicator for the absence of the parasite in bryozoans in the same location. This disease is extremely complex and there are several variables which might decouple infection in the two hosts.

If I am reading the methods correctly, only fish from the Wutach were pit-tagged, which means that there is no movement data for fish that were above the barrier before its removal. These fish may have very different patterns of movement across the two water bodies. This missing information represents a large gap for any epidemiological model of this system.

Specific Comments:

Line 58: “…still a point of concern~~s~~.” De-pluralize to “concern”.

Line 68: “primary hosts” is probably a better term for bryozoans than “final hosts”

Line 69 and throughout: “actinospore” and “myxospore” are terms specifically applied to organisms from the class myxosporea (e.g. M. cerebralis), rather than those of malacosporea (e.g. *T. bryosalmonae*). The best terms that we have for malacosporean spores are along the lines of “fish malacospores”, for those infectious to fish, and “bryozoan malacospores” for those infectious to bryozoans, etc.

Line 76: “with a peak in disease severity and myxospore release in late summer / early autumn (30).” Bailey et al. 2017 seems to be a reference for the disease severity part of the statement, but not for the myxospore release part. Tops et al. 2006 is probably the best citation for the later. Note however that neither study is directly linking seasonality. In both cases temperature, is the independent variable. I suggest changing the language to more accurately reflect that nuance, something like “with peaks in disease severity (Bailey et al. 2017) and myxospore release (Tops et al. 2006) corresponding with high temperatures typical of late summer/ early fall.”

Tops, S., Lockwood, W. and Okamura, B., 2006. Temperature-driven proliferation of Tetracapsuloides bryosalmonae in bryozoan hosts portends salmonid declines. Diseases of Aquatic Organisms, 70(3), pp.227-236.

Line 77: You could cite Gay et al. 2001 or Tops et al. 2006 here.

Gay, M., Okamura, B. and De Kinkelin, P., 2001. Evidence that infectious stages of Tetracapsula bryosalmonae for rainbow trout Oncorhynchus mykiss are present throughout the year. Diseases of Aquatic Organisms, 46(1), pp.31-40.

Figure 1: The satellite imagery base map makes this figure very hard to see/interpret. The red and black text are especially difficult to read. I would rather see a simple base map with water bodies and sample locations depicted.

Line 143: should read “18s rDNA” instead of “18 rDNA”

Throughout methods: Please describe your control schemes in more detail. Were there any extraction controls? If so, how many, with what frequency, and what material was used? Same for qPCR negative controls please.

Figure 2: This image does not seem necessary.

Line 169: Did you multiplex antennae to determine directionality of fish movement across the array? This would be a good detail to know. The language right now suggests that it was a singleplex antenna array.

Line184: Are there any specific packages or macros that need to be referenced? Methods are intended to be repeatable, but this “statistics” sub-section only mentions that some software was used. What tests were performed? I also did not see any reference to statistical tests in the results or discussion sections.

Line191: Maybe this is more of an editorial issue, but I think this should read “… according to the criteria of Froese et al. 2016 (32).”

We lost line numbers after line 191.

“In brown trout originating from the Ehrenbach, in none of the examined fish at any time, neither macroscopic nor histological signs of an infection with T. bryosalmonae were found.”

“originating” seems like a poor word to use here. I think you mean “sampled”. Otherwise, what is meant by “originating”?

In the “Water Temperature” sub-section the font style seems to change.

“During the cold season excretion of T. bryosalmonae spores by brown trout does not play a role.”

I don’t think that this sentence accurately describes anything in particular. It should be revised or removed.

Throughout the manuscript: All of the text should be reviewed by someone who is well versed in English grammar.
