# Supplementary material for: The role of migration barriers for dispersion of Proliferative Kidney Disease—Balance between disease emergence and habitat connectivity
Source: PLoS One. 2021 Mar 17;16(3):e0247482. doi: 10.1371/journal.pone.0247482 (PMC7968629; doi:10.1371/journal.pone.0247482)
Supplement: Attachment — Submitted filename: 2020_PLOS1_ms_Migration barriers_PKD_HIGHLIGHTS.docx [file pone.0247482.s002.docx]

**The role of migration barriers for dispersion of Proliferative Kidney Disease – balance between disease emergence and habitat connectivity**

Running title: Role of migration barriers for dispersion of PKD

Heike Schmidt-Posthaus^1*^, Ernst Schneider^2^, Regula Hirschi^1^, Nils Schölzel^3^, Armin Peter^3^

^1^Centre for Fish and Wildlife Health, Department of Infectious Diseases and Pathobiology, Vetsuisse Faculty, University of Bern, Länggassstrasse 122, 3012 Bern, Switzerland

^2^Alte Landstrasse 156, 8800 Thalwil, Switzerland

^3^ FishConsulting GmbH, Hagmattstrasse 7, 4600 Olten, Switzerland

***CORRESPONDING AUTHOR**: Heike Schmidt-Posthaus, Centre for Fish and Wildlife Health, Department of Infectious Diseases and Pathobiology, Vetsuisse Faculty, University of Bern, Länggassstrasse 122, 3012 Bern, Switzerland, +41 (0)31 631 2465 , [heike.schmidt@vetsuisse.unibe.ch](mailto:heike.schmidt@vetsuisse.unibe.ch)

**Summary**

Natural and uninterrupted water courses are important for biodiversity and fish population stability. Nowadays, many streams and rivers are obstructed and artificial migration barriers are often preventing the migration of fish. On the other hand, distribution of pathogens by migrating fishes is still a point of concerns. Pathogen transport and transmission is a driving force in the dynamics of many infectious diseases. The aim of the study was to investigate the role of an artificial migration barrier in limitation of pathogen transport by migrating fish, in this case of Proliferative Kidney Disease (PKD) in brown trout. To test this question, a river system was selected with a migration barrier separating a PKD positive river from a PKD negative tributary. After removal of the barrier, PKD prevalence and pathology was examined during five years after elimination of the barrier. To confirm upstream migration of brown trout, PIT tagging of wild trout was used. Adult brown trout were migrating upstream during winter months, presumably for spawning. Despite this migration no PKD positive fish were detected during the examination period.

Our study illustrates the importance of the connectivity of streams for habitat maintenance and productivity. Although migration of brown trout from a PKD-positive river into a PKD-negative tributary was confirmed, upstream spreading of PKD was not observed. Therefore, the benefit of restoration seems to exceed the risk of transmission of infectious agents at least over limited periods.

Keywords: Migration barrier, Proliferative Kidney Disease, restoration, habitat maintenance

**Introduction**

Water courses are important as part of naturally formed landscapes as well as a vital part of developing ecosystems. Nowadays, many streams and rivers are obstructed and migration barriers are often preventing free migration of fishes. For decades, free flowing rivers in Central Europe were seen as a threat rather than as a benefit. Thus, despite the fact that such areas are the only functioning or potential reservoirs of biodiversity. Fragmentation of river networks can increase the isolation of fish populations (1–4). Species like brown trout (Salmo trutta,) or salmon (*Salmo salar*) are particularly sensitive to barriers, as juvenile and adult life stages must make extensive migrations within freshwater river systems what is particularly relevant for the salmon Barriers are therefore a potentially important constraint on production and population stability where access to and from spawning and rearing habitats is limited, delayed, or even barred, e.g. (5). Nowadays, the restoration of the water courses is included as a dynamic part of several current projects, which propose full connectivity for migrating fish, expansion of water space and ability of fishes to migrate (Federal Office for the Environment, pers. comm.).

On the other hand, distribution of pathogens by migrating fishes is still a point of concerns. Pathogen transport and transmission is a driving force in the dynamics of many infectious diseases (6). Proliferative Kidney Disease (PKD) is one example, where discussion arose of weighting interests between habitat upgrade and dispersion of devastating diseases. PKD was classified as an emerging disease in Europe (7) with high losses in farmed rainbow trout (*Oncorhynchus mykiss*) (8,9) and wild salmonid populations (10–12). It was discussed as one factor for the long term population decline of wild brown trout (*Salmo trutta*) in many Swiss midland river systems (13) and in neighboring Southern Germany (14). PKD is caused by *Tetracapsuloides bryosalmonae,* belonging to the Malacospora, which were assigned to the phylum Cnidaria (15–19). The life cycle of *T. bryosalmonae* involves bryozoans, mainly *Fredericella sultana*, as invertebrate or final host (17,20,21) and salmonids as vertebrate or intermediate host (16,22). Infected bryozoans release spores, i.e. actinospores that infect fish, mainly salmonids, by penetrating the gill epithelium and vascular walls. Through haematogenic dispersion a generalized infection occurs, with the kidney as main target organ (23). There the parasite develops into a myxospore, which migrates into the tubular lumen and is eventually excreted via the urine (24,25). These myxospores infect susceptible bryozoans (26–28). In salmonids, mainly young-of-the-year (YOY) trout are affected, when animals got in contact to the parasite for the first time in their life (7). PKD is a temperature dependent disease (25,29), with a peak in disease severity and myxospore release in late summer / early autumn (30). During winter and spring, the infection is usually inactive and asymptomatic.

The aim of the study was to investigate the role of a migration barrier in limitation of pathogen transport by migrating fish, in this case of PKD in brown trout. To test this question, a river system was selected with a migration barrier separating a PKD positive river, the Wutach, from a PKD negative tributary, the Ehrenbach. The removed barrier was situated close to the mouth of the Ehrenbach into the Wutach.

PKD prevalence and pathology was examined immediately before and during five years after elimination of the barrier. To confirm upstream migration of brown trout into the Ehrenbach, PIT tagging of wild trout from the Wutach was used.

**Material and Methods**

**Ethics statement**

This study was carried out in accordance with the University of Bern Animal Ethics Committee. Approval for animal experiments was obtained from the Cantonal Veterinary Offices in Bern, Zürich and Schaffhausen, Switzerland (Authorization # BE102/14+ and # 75698).

**Study sites and fish sampling**

The river Wutach and its tributary Ehrenbach are situated at the border between Southern Germany and the North-eastern part of Switzerland (Fig. 1). The selection of the river system was based on former investigations, showing no PKD in brown trout in the Ehrenbach, but high infection prevalence in brown trout in the Wutach, ranging from 90 to 100% (Schmidt-Posthaus et al. 2015; own investigations). Beginning in 2013 (14^th^ of September), 20 brown trout were sampled above (Ehrenbach) and below the migration barrier (Wutach) to monitor the PKD status, as a zero sampling. Migration barrier was eliminated in 2014. Starting from 2016 until 2019, PKD status was evaluated. Samples were collected annually by electrofishing, same day from the Wutach and the Ehrenbach, between 24^th^ of August and 15^th^ of September.

**Fig 1. Map of study area.** Locations of electrofishing, PIT tagging, monitoring antenna and temperature loggers are shown.

Brown trout were captured by electrofishing. Two stretches of 100 m in the river Wutach (2’677’061.2/1’289’694.7, 2’677’814.2/1’291’059.7) and one of 50 m in the Ehrenbach (2’674’786/1’292’254), approximately 4 km upstream from the migration barrier, or the removed barrier, respectively (Fig. 1), were investigated. Twenty YOY brown trout from each stream were sampled and euthanized separately by 100 mg/L buffered tricaine methanesulfonate (MS 222®, Argent Chemical Laboratories, Redmont, USA).

**Pathology and histopathology**

Length and weight of every euthanized fish was recorded and the condition factor was calculated (100*weight/lenght^3^, Bagenal 1978, Froese & Pauly 2016). A complete necropsy was performed. Macroscopic changes in the inner organs were evaluated. Kidneys were removed, and cut longitudinally. One part was immediately fixed in 10 % buffered formalin for histopathological examination. The other half was fixed in RNAlater® (DNA stabilisation solution) for real time PCR analysis. At the Centre for Fish and Wildlife Health (CFWH), formalin fixed samples were routinely prepared for histology and stained with haematoxylin and eosin (HE). HE stained slides were examined by light microscopy (Nikon Eclipse E400Nikon). In renal histopathology, the following criteria were considered: proliferation of interstitial tissue, vasculitis with thrombi, interstitial haemorrhage, vessel wall necrosis and inflammation with mainly macrophages and lymphocytes, interstitial necrosis, interstitial inflammation predominantly with macrophages, interstitial fibrosis, tubulonephrosis and tubuloneogenesis (29). These histopathological changes were graded as 0 (no) to 6 (severe). Presence of *T. bryosalmonae* was examined on one histological section through the entire kidney and infection intensity was classified ranging from 0 (no parasites present on whole slide) to 6 (high numbers of parasites in renal hematopoietic tissue, vessels and / or tubules) at a magnification of 200 to 400x.

**qPCR for detection of *T. bryosalmonae* DNA in kidney tissue**

Fish negative or suspicious for *T. bryosalmonae* by histology were analysed by qPCR as described by (31). Before extraction, kidneys were homogenized in a 2 ml tube containing 1.5 ml Tri-solution (Sigma, Switzerland) with a 2 mm diameter steel bead (QIAGEN, Switzerland) using a tissue lyser (QIAGEN, Switzerland) with a shaking frequency set at 30 shakes per second for 3 min. Genomic DNA was extracted by using a DNeasy tissue Kit (Quiagen, Basel, Switzerland) according to the manufacturer’s instructions. DNA was finally eluted in 30 µl of EB buffer (QIAGEN, Switzerland) and stored at -20°C until qPCR was performed. qPCR was performed targeting *T. bryosalmonae* 18 rDNA (Acc. N.: AF190669) specific TaqMan method according to Bettge et al. (2009a), using the following primers and probe, PKDtaqf1: 5′–GCGAGATTTGTTGCATTTAAAAAG– 3′and PKDtaqr1: 5′ –GCACATGCAGTGTCCAA TCG– 3′), probe PKD (5′–CAAAATTGTGGAACCGTCCGACTACGA–3′). All reactions were carried out in duplicates. Non-target controls (DNAse free water) within the qPCR never showed amplification, while the internal controls (Exo IPC) were always amplified, showing no qPCR inhibition.

**Monitoring of migration by tagging wild trout**

For migration monitoring, on 24^th^ of August 2017, electrofishing was done at two sites in the river Wutach, at a length of 280 m each. One location (start 2’677’061.2/1’289’694.7, end 2’677’155.2/1’289’950.7) was situated 1200 m downstream of the inflow of the Ehrenbach, the other location (start 2’677’814.2/1’291’059.7, end 2’678’012.2/1’291’261.7) was situated 280 m upstream the inflow. 162 brown trout and two rainbow trout were captured, 68 brown trout at the upper location, 96 fish (94 brown trout, 2 rainbow trout) at the downstream location.

Fish were anesthetized with 1 ml clove oil (80-95% Eugenol®) in aerated 30 l water, the body cavity was opened by a 3-4 mm incision and a half-duplex (HDX) PIT - tag (OREGON RFID, Portland, USA) was implanted in every fish for individual recognition. Depending on the size of the fish, a 12 mm (fish < 150 mm) or 23 mm PIT-tag (fish > 150 mm) was implanted (Fig. 2). After implantation, fish were kept for at least 30 min in an aerated tank (100x50x30 cm) for recovery. Afterwards, trout were released into the respective river stretch.

**Fig. 2. PIT-tags which were implanted into body cavity**. Depending on size of fish, a 12 mm or 23 mm PIT-tag was implanted, coin diameter 23.2 mm

For detection of migrating fish, a HDX-antenna with an automatic registration and single reader (OREGON RFID, Portland, USA) was installed 300 m upstream of the mouth of Ehrenbach, (2’677’531.8/1’291’207.6). Migrating fish were registered according to the electromagnetic field, fish ID and time point were registered on every passage.

With single daily interruptions due to flooding’s or technical problems, the antenna was kept functioning until January 2020.

**Water temperature**

Water temperature from Ehrenbach (2’674’615/1’292’318) and Wutach (2’676’398/1’289’323) was recorded between 2013 and 2018, from April to October every 2 hours by Onset TidbiT v2 Temp Loggers (Fig. 1). For 2019, no data were available from Ehrenbach, because the logger was lifted. Mean values for each day were calculated and plotted.

**Statistics**

Migration data were analysed using *R* 3.1.2 (R Core Team 2018) as well as Excel 2010.

Significance level was set at p≤0.05.

**Results**

**Fish parameters**

Length, weight and condition index of brown trout samples from Wutach and Ehrenbach between 2013 and 2019 are shown in table 1. Over the years, condition index varied within normal limits for brown trout, according to the criteria of (32).

**Table 1. Fish body parameter.**

Number (n) of sampled fish, length, weight and condition index ~~of brown trout~~. Shown are range, mean and standard deviation of length, weight and condition index. Range is shown in parenthesis. nd = no data

|  | Ehrenbach | | | | | Wutach | | | | | |
| --- | --- | --- | --- | --- | --- | --- | --- | --- | --- | --- | --- |
|  | 2013 | 2016 | 2017 | 2018 | 2019 | 2013 | 2016 | 2017 | 2018 | 2019 |  |
| n | 15 | 27 | 30 | 20 | 20 | 11 | 22 | 26 | 12 | 20 |  |
| Length (mm) | 83±15  (79-135) | 99±19.2  (80-160) | 99±8.67  (80-160) | 99±15.31  (78-135) | 90±14.14  (66-105) | 102±9.6  (85-115) | 102±13  (80-130) | 101±15.04  (80-130) | 129±28.34  (100-195) | 98±12.64  (68-120) |  |
| Weight (g) | nd | 12±8  (5-38) | 12±7.23  (5-38) | 11.2±5.28  (6-25) | 7.8±2.42  (3-12) | nd | 11.8±4.38  (6-22) | 11.8±5.12  (6-22) | 25±18.73  (11-72) | 10.5±3.94  (4-21) |  |
| Condition index | nd | 1.19±0.14  (0.9-1.47) | 1.19±0.14  (0.9-1.47) | 1.13±0.32  (0.9-2.3) | 1.03±0.11  (0.77-1.21) | nd | 1.09±0.12  (0.85-1.3) | 1.09±0.12  (0.85-1.3) | 1.01±0.09  (0.8-1.1) | 1.09±0.2  (0.73-1.63) |  |

**PKD status**

At the first sampling in 2013, before removal of the migration barrier, 15 brown trout sampled in the Ehrenbach showed no signs of PKD, neither macroscopically nor histologically. By qPCR, no *T. bryosalmonae* DNA was detected. In the Wutach, 9 out of 11 animals (81.8%) showed histological signs of an infection with *T. bryosalmonae*. Eight animals showed acute lesions with vascular thrombosis, interstitial necrosis and hemorrhage, interstitial infiltration with lymphocytes and macrophages and low to high number of parasites (table 3). One animal showed chronic active changes with interstitial fibrosis and concurrent acute signs as described above. Severity of changes and parasite abundance are summarized in table 3.

2014, the migration barrier was removed. To identify a possible invasion of the parasite from the PKD positive Wutach into the PKD negative Ehrenbach via migrating brown trout, annual samplings were implemented from 2016 to 2019. In brown trout originating from the Ehrenbach, in none of the examined fish at any time, neither macroscopic nor histological signs of an infection with *T. bryosalmonae* were found. All animals from the Ehrenbach were additionally investigated by qPCR. In 2016 in one brown trout, *T. bryosalmonae* DNA was identified (Ct values (duplicate measurements): 33.6 and 31.7). This result was confirmed by sequencing. The product showed a 100% identity to the *T. bryosalmonae* 18S ribosomal RNA gene (Accession n° EU570235.1). All other qPCR investigations were negative (table 3).

From 2016 to 2019, 50 to 92% of investigated brown trout showed histological signs of PKD with acute lesions and interstitial extrasporogonic parasite stages (table 2). Several specimens showed intratubular myxospores. By qPCR, all remaining brown trout revealed to be positive for *T. bryosalmonae* DNA with a total prevalence of 100% during the investigation period (table 3).

**Table 2. Prevalence, parasite abundance and associated pathology of *T. bryosalmonae* infection in brown trout in Ehrenbachh and Wutach in 2013 and from 2016 to 2019**

Shown are mean values and standard deviation (SD) for parasite abundance and pathology severity grades

| River system | year | n | Prevalence (%) | | Parasite abundance  (mean±SD) | Pathology severity (mean±SD) |
| --- | --- | --- | --- | --- | --- | --- |
|  |  |  | Histology | qPCR  (Ct values) |  |  |
| Ehrenbach | 2013 | 15 | 0 | 0 | 0 | 0 |
|  | 2016 | 27 | 0 | 4*  (31.7 / 33.6) | 0 | 0 |
|  | 2017 | 30 | 0 | 0 | 0 | 0 |
|  | 2018 | 20 | 0 | 0 | 0 | 0 |
|  | 2019 | 20 | 0 | 0 | 0 | 0 |
| Wutach | 2013 | 11 | 82 | nd | 2.8±1.3 | 3.6±1.9 |
|  | 2016 | 22 | 77 | 100  (23.85 - 37.65) | 2.1±1.5 | 3±1.8 |
|  | 2017 | 26 | 75 | 100  (19.56 - 24.2) | 2.7±1.5 | 3.6±1.6 |
|  | 2018 | 12 | 92 | 100  (39.14) | 2.8±1.5 | 3.5±1.5 |
|  | 2019 | 20 | 50 | 100  (19.98 - 34.58) | 1.9±1.4 | 2.1±1.5 |

*one positive animal

**Fish migration**

162 brown trout and two rainbow trout were PIT tagged. Length of tagged fish ranged from 90 to 471 mm (mean 270.9, median 282), weight from 9 to 1072 (mean 296.6, median 269.5) g. Condition index ranged from 0. 85 to 1.78 (mean 1.19).

On 15^th^ of December 2017, the first migrating brown trout was identified in the Ehrenbach. Monitoring was continued until 6^th^ of January 2020. Migration of brown or rainbow trout was only detected between end of November and beginning of April of the respective years. During the investigation period, 19 tagged brown trout and one rainbow trout were migrating from the Wutach into the Ehrenbach (12%) (Fig. 3). 143 tagged brown trout and one tagged rainbow trout were not detected (Fig. 3). Fish below 150 mm were never detected in the Ehrenbach.

**Fig. 3. Histogram of animals migrating into the Ehrenbach.** Length of animals which is shown in figure is the legth of the fish at the time point of tagging. Observations took place over two years. Light grey bars = brown trout tagged in upstream stretch in Wutach, grey bars = brown trout tagged in downstream stretch in Wutach, dark grey bar = rainbow trout

**Water temperature**

In 2013, water temperature was higher in the Wutach compared to the Ehrenbach. However, in Ehrenbach, water temperature was increasing during the investigation period, levelling similar to the temperature measured in the Wutach (Figure 4). In the Ehrenbach, water temperature exceeded 15°C over 8 non-consecutive days in 2013 (3 days in July and 5 day in August), over 65 non-consecutive days in 2017 (July till August) and over 51 non-consecutive days in 2918 (July till August). In the Wutach, water temperature exceeded 15°C over 48 non-consecutive days in 2013 (July till August), over 83 non-consecutive days in 2017 (June till August) and over 80 non-consecutive days in 2918 (June till August).

**Fig. 3. Temperature profiles from Ehrenbach and Wutach, 2013, 2017 and 2018.**

Red line marks 15°C, the temperature where PKD revealed to be clinically relevant

**Discussion**

River regulation for flood protection purposes and the construction of barriers for hydropower generation, or irrigation for agricultural production has led to a numerical increase of anthropogenically impacted water bodies. However, nowadays restoration becomes more and more significant. Migrating fish populations are strongly affected by in- and inter-stream barriers that cause river network fragmentation, constraining productivity or preventing completion of their lifecycle. Removal or reduction of barrier impacts is a restoration measure associated with unambiguous benefits. So, for brown trout, which migrate considerable distances, free connection of river systems is essential to reach spawning habitats. In addition, increased connectivity also increases biodiversity facilitating migration also for smaller fish species (33) and invertebrates. On the other hand, spreading of infectious agents possibly detrimental for vulnerable fish populations can be enhanced by migrating fish.

PKD is one example of an emerging disease rapidly spreading throughout Europe during the last decades (10,12,34). This spreading is due to a complex interaction of multiple factors (12,25,29,35–37). It was shown that distribution of PKD is related to the distribution of bryozoan, the final host in the parasite cycle (38). For the Ehrenbach the presence of *Fredericella sultana* is not confirmed yet. The temperature profile of the rivers Wutach and Ehrenbach showed that water temperature was higher in the PKD positive Wutach. However, also in the Ehrenbach, water temperature exceeded 15°C for several days in a row every year. 15°C is accepted as the permissive temperature for development of a clinically relevant PKD (29,39). Even if the minimum necessary number of days with high water temperature for the clinical outbreak of disease is not known and possibly differs in conjunction with other environmental factors, water temperature alone as an excluding factor for parasite distribution upstream can be excluded. Migration from the Wutach into the Ehrenbach, was mainly seen in winter months, from December to April. This supports the hypothesis that the Ehrenbach is an important spawning stream for brown trout. During the cold season excretion of *T. bryosalmonae* spores by brown trout does not play a role. Its peak occurs mainly in late summer (25,40). In addition, PKD is a disease primarily of YOY, an age class not migrating upstream for spawning. In the first year after barrier removal, PCR showed *T. bryosalmonae* DNA in one single fish. This was as a unique, isolated finding over the whole survey period, not associated with disease, tissue changes or parasite detection.

Considering the technical malfunction of the PIT antenna over cumulated 91 days, between October 2017 and March 2018 (i. e. mainly during the spawning migration period) due to flooding and electrical supply failure, the number of registered events might not reflect the accurate number of all migration movements. Nevertheless, the total detection rate over 27 months of tagged brown and rainbow trout migrating into the Ehrenbach reached 12% (20 out of 164). Only adult fish were detected. None of the 21 tagged YOY fish were registered in the Ehrenbach. This might have multiple reasons, but upstream areas were used by adult brown trout only for spawning, what migration patterns confirm. Mortality due to PKD mainly affects YOY (16). Even if exact data are difficult to generate under field conditions, associated PKD mortality in the Wutach seems to not exceed 15% in brown trout, shown in exposition experiments in the past (41,42).

**Conclusion**

Our study illustrates that, after removal of migration barrier, adult brown trout were migrating upstream during winter months. This proves the importance of continuity of this stream for habitat maintenance and productivity. Although migration of brown trout from a PKD-positive river into a PKD-negative tributary was confirmed, upstream spreading of the disease was not observed over a five years period. Therefore, the benefit of restoration of the river corridor seems to exceed the risk of transmission of infectious agents at least over limited periods.

**Acknowledgements**

We thank the fishermen „Oberes Wutachtal Stühlingen e.V.“ for help during electrofishing and the staff of the histology laboratory of the Institute of Animal Pathology for preparation of the histological sections.

**FINANCIAL DISCLOSURE STATEMENT**

The study was financially supported by the Federal Office for the Environment (FOEN), Bern, Switzerland (HS, AP) (www.bafu.admin.ch). The funders had no role in study design, data collection and analysis, decision to publish, or preparation of the manuscript.

**CoNFLICT OF interests**

The authors declare no conflict of interests.

**DATA AVAILABILITY**

Data are available upon request by the authors

**REFERENCES**

1.	Campbell Grant EH, Lowe WH, Fagan WF. Living in the branches: population dynamics and ecological processes in dendritic networks. Ecol Lett 2007;10(2):165–75. doi: 10.1111/j.1461-0248.2006.01007.x. PubMed PMID: 17257104.

2.	Freeman MC, Bowen ZH, Bovee KD, Irwin ER. Flow and Habitat Effects on Juvenile Fish Abundance in Natural and Altered Flow Regimes. Ecological Applications 2001;11(1):179. doi: 10.2307/3061065.

3.	Junge C, Museth J, Hindar K, Kraabøl M, Vøllestad LA. Assessing the consequences of habitat fragmentation for two migratory salmonid fishes. Aquatic Conserv: Mar. Freshw. Ecosyst. 2014;24(3):297–311. doi: 10.1002/aqc.2391.

4.	SCHICK RS, LINDLEY ST. Directed connectivity among fish populations in a riverine network. Journal of Applied Ecology 2007;44(6):1116–26. doi: 10.1111/j.1365-2664.2007.01383.x.

5.	Venditti DA, Rondorf DW, Kraut JM. Migratory Behavior and Forebay Delay of Radio-Tagged Juvenile Fall Chinook Salmon in a Lower Snake River Impoundment. North American Journal of Fisheries Management 2000;20(1):41–52. doi: 10.1577/1548-8675(2000)020%3C0041:MBAFDO%3E2.0.CO;2.

6.	McCallum H. How should pathogen transmission be modelled? Trends in Ecology & Evolution 2001;16(6):295–300. doi: 10.1016/S0169-5347(01)02144-9.

7.	Okamura B, Hartikainen H, Schmidt-Posthaus H, Wahli T. Life cycle complexity, environmental change and the emerging status of salmonid proliferative kidney disease. Freshwater Biology 2011;56(4):735–53. doi: 10.1111/j.1365-2427.2010.02465.x.

8.	Feist SW. Progress on proliferative kidney disease (PKD) research. Trout News 2004(38):17–9.

9.	Palikova M, Papezikova I, Markova Z, Navratil S, Mares J, Mares L, et al. Proliferative kidney disease in rainbow trout (Oncorhynchus mykiss) under intensive breeding conditions: Pathogenesis and haematological and immune parameters. Vet Parasitol 2017;238:5–16. doi: 10.1016/j.vetpar.2017.03.003. PubMed PMID: 28291603.

10.	Kristmundsson Á, Antonsson T, Árnason T. First record of Proliferative Kidney Disease in Iceland. Bulletin of the European Association of Fish Pathologists 2010(30 (1)):35–40.

11.	Skovgaard A, Buchmann K. Tetracapsuloides bryosalmonae and PKD in juvenile wild salmonids in Denmark. Dis Aquat Org 2012;101(1):33–42. doi: 10.3354/dao02502. PubMed PMID: 23047189.

12.	Wahli T, Knuesel R, Bernet D, Segner H, Pugovkin D, Burkhardt-Holm P, et al. Proliferative kidney disease in Switzerland: current state of knowledge. J Fish Diseases 2002;25(8):491–500. doi: 10.1046/j.1365-2761.2002.00401.x.

13.	Burkhardt-Holm P, Giger W, Güttinger H, Ochsenbein U, Peter A, Scheurer K, et al. Where have all the fish gone? The reasons why fish catches in Swiss rivers are declining. Environ Sci Technol 2005;39(21):441A-447A. doi: 10.1021/es053375z. PubMed PMID: 16294843.

14.	Ros A, Basen T, Schneider E, Schmidt-Posthaus H. Die Verbreitung einer temperaturempfindlichen Nierenerkrankung PKD in Bachforellen in Baden-Württemberg. AUF AUF 2018(1):40–3.

15.	Canning EU, Curry A, Feist SW, Longshaw M, Okamura B. A new class and order of myxozoans to accommodate parasites of bryozoans with ultrastructural observations on Tetracapsula bryosalmonae (PKX organism). J Eukaryot Microbiol 2000;47(5):456–68. doi: 10.1111/j.1550-7408.2000.tb00075.x. PubMed PMID: 11001143.

16.	Hedrick RP, MacConnell E, Kinkelin P de. Proliferative kidney disease of salmonid fish. Annual Review of Fish Diseases 1993;3:277–90. doi: 10.1016/0959-8030(93)90039-E.

17.	Okamura B, Anderson CL, Longshaw M, Feist SW, Canning EU. PATTERNS OF OCCURRENCE AND 18S rDNA SEQUENCE VARIATION OF PKX (TETRACAPSULA BRYOSALMONAE ), THE CAUSATIVE AGENT OF SALMONID PROLIFERATIVE KIDNEY DISEASE. Journal of Parasitology 2001;87(2):379–85. doi: 10.1645/0022-3395(2001)087[0379:POOARS]2.0.CO;2.

18.	Jiménez-Guri E, Philippe H, Okamura B, Holland PWH. Buddenbrockia is a cnidarian worm. Science 2007;317(5834):116–8. doi: 10.1126/science.1142024. PubMed PMID: 17615357.

19.	Nesnidal MP, Helmkampf M, Bruchhaus I, El-Matbouli M, Hausdorf B. Agent of whirling disease meets orphan worm: phylogenomic analyses firmly place Myxozoa in Cnidaria. PLoS ONE 2013;8(1):e54576. doi: 10.1371/journal.pone.0054576. PubMed PMID: 23382916; PubMed Central PMCID: PMC3559788.

20.	Anderson CL, Canning EU, Okamura B. 18S rDNA sequences indicate that PKX organism parasitizes Bryozoa. Bulletin of the European Association of Fish Pathologists 1999(19 (3)):94–7.

21.	Longshaw M, Feist SW, Canning EU, Okamura B. First identification of PKX in bryozoans from the United Kingdom – Molecular evidence. Bulletin of the European Association of Fish Pathologists 1999(19):146–8.

22.	Feist SW, Bucke D. Proliferative kidney disease in wild salmonids. Fisheries Research 1993;17(1-2):51–8. doi: 10.1016/0165-7836(93)90006-S.

23.	Feist SW, Longshaw M, Canning EU, Okamura B. Induction of proliferative kidney disease (PKD) in rainbow trout Oncorhynchus mykiss via the bryozoan Fredericella sultana infected with Tetracapsula bryosalmonae. Dis Aquat Org 2001;45(1):61–8. doi: 10.3354/dao045061. PubMed PMID: 11411645.

24.	Hedrick RP, Baxa DV, Kinkelin P de, Okamura B. Malacosporean-like spores in urine of rainbow trout react with antibody and DNA probes to Tetracapsuloides bryosalmonae. Parasitol Res 2004;92(1):81–8. doi: 10.1007/s00436-003-0986-3. PubMed PMID: 14610667.

25.	Strepparava N, Segner H, Ros A, Hartikainen H, Schmidt-Posthaus H, Wahli T. Temperature-related parasite infection dynamics: the case of proliferative kidney disease of brown trout. Parasitology 2018;145(3):281–91. doi: 10.1017/S0031182017001482. PubMed PMID: 28831940.

26.	Grabner DS, El-Matbouli M. Transmission of Tetracapsuloides bryosalmonae (Myxozoa: Malacosporea) to Fredericella sultana (Bryozoa: Phylactolaemata) by various fish species. Dis Aquat Org 2008;79(2):133–9. doi: 10.3354/dao01894. PubMed PMID: 18500029.

27.	Morris DJ, ADAMS A. Transmission of Tetracapsuloides bryosalmonae (Myxozoa: Malacosporea), the causative organism of salmonid proliferative kidney disease, to the freshwater bryozoan Fredericella sultana. Parasitology 2006;133(Pt 6):701–9. doi: 10.1017/s003118200600093x. PubMed PMID: 16948873.

28.	Tops S, Baxa DV, McDowell TS, Hedrick RP, Okamura B. Evaluation of malacosporean life cycles through transmission studies. Dis Aquat Org 2004;60(2):109–21. doi: 10.3354/dao060109. PubMed PMID: 15460855.

29.	Bettge K, Wahli T, Segner H, Schmidt-Posthaus H. Proliferative kidney disease in rainbow trout: time- and temperature-related renal pathology and parasite distribution. Dis Aquat Org 2009;83(1):67–76. doi: 10.3354/dao01989. PubMed PMID: 19301638.

30.	Bailey C, Segner H, Casanova-Nakayama A, Wahli T. Who needs the hotspot? The effect of temperature on the fish host immune response to Tetracapsuloides bryosalmonae the causative agent of proliferative kidney disease. Fish Shellfish Immunol 2017;63:424–37. doi: 10.1016/j.fsi.2017.02.039. PubMed PMID: 28238860.

31.	Bettge K, Segner H, Burki R, Schmidt-Posthaus H, Wahli T. Proliferative kidney disease (PKD) of rainbow trout: temperature- and time-related changes of Tetracapsuloides bryosalmonae DNA in the kidney. Parasitology 2009;136(6):615–25. doi: 10.1017/S0031182009005800. PubMed PMID: 19366483.

32.	Froese R, Pauly D. FishBase. World Wide Web electronic publication [Internet]; 2016. Available from: www.fishbase.org, version.

33.	Shao X, Fang Y, Jawitz JW, Yan J, Cui B. River network connectivity and fish diversity. Science of The Total Environment 2019;689:21–30. doi: 10.1016/j.scitotenv.2019.06.340.

34.	Waldner K, Bechter T, Auer S, Borgwardt F, El‐Matbouli M, Unfer G. A brown trout (Salmo trutta ) population faces devastating consequences due to proliferative kidney disease and temperature increase: A case study from Austria. Ecol Freshw Fish 2019. doi: 10.1111/eff.12528.

35.	Bailey C, Rubin A, Strepparava N, Segner H, Rubin J-F, Wahli T. Do fish get wasted? Assessing the influence of effluents on parasitic infection of wild fish. PeerJ 2018;6:e5956. doi: 10.7717/peerj.5956. PubMed PMID: 30479904; PubMed Central PMCID: PMC6238765.

36.	Dash M, Vasemägi A. Proliferative kidney disease (PKD) agent Tetracapsuloides bryosalmonae in brown trout populations in Estonia. Dis Aquat Org 2014;109(2):139–48. doi: 10.3354/dao02731. PubMed PMID: 24991741.

37.	Gorgoglione B, Kotob MH, Unfer G, El-Matbouli M. First Proliferative Kidney Disease outbreak in Austria, linking to the aetiology of Black Trout Syndrome threatening autochthonous trout populations. Dis Aquat Org 2016;119(2):117–28. doi: 10.3354/dao02993. PubMed PMID: 27137070.

38.	Carraro L, Bertuzzo E, Mari L, Fontes I, Hartikainen H, Strepparava N, et al. Integrated field, laboratory, and theoretical study of PKD spread in a Swiss prealpine river. Proc Natl Acad Sci U S A 2017;114(45):11992–7. doi: 10.1073/pnas.1713691114. PubMed PMID: 29078391; PubMed Central PMCID: PMC5692590.

39.	Wahli T, Bernet D, Segner H, Schmidt-Posthaus H. Role of altitude and water temperature as regulating factors for the geographical distribution of Tetracapsuloides bryosalmonae infected fishes in Switzerland. Journal of Fish Biology 2008;73(9):2184–97. doi: 10.1111/j.1095-8649.2008.02054.x.

40.	Schmidt-Posthaus H, Steiner P, Müller B, Casanova-Nakayama A. Complex interaction between proliferative kidney disease, water temperature and concurrent nematode infection in brown trout. Dis Aquat Org 2013;104(1):23–34. doi: 10.3354/dao02580. PubMed PMID: 23670077.

41.	Schmidt-Posthaus H, Ros A, Hirschi R, Schneider E. Comparative study of proliferative kidney disease in grayling Thymallus thymallus and brown trout Salmo trutta fario: an exposure experiment. Dis Aquat Org 2017;123(3):193–203. doi: 10.3354/dao03102. PubMed PMID: 28322206.

42.	Schmidt-Posthaus H, Hirschi R, Schneider E. Proliferative kidney disease in brown trout: infection level, pathology and mortality under field conditions. Dis Aquat Org 2015;114(2):139–46. doi: 10.3354/dao02855. PubMed PMID: 25993888.
